# Placental defects revealed by modelling Prader–Willi syndrome in mice

**DOI:** 10.1242/dmm.052833

**Published:** 2026-09-01

**Authors:** Anna E. Webberley, Raquel Boque-Sastre, Lauren Bailey, Cerys Charles, Rachel A. Jones, Rosie Bunton-Stasyshyn, Michelle E. Stewart, Sara E. Wells, David S. Chatelet, Stephanie K. Robinson, Matthew J. Higgs, Rosalind M. John, Anthony R. Isles

**Affiliations:** ^1^ Centre for Neuropsychiatric Genetics and Genomics, Division of Psychological Medicine and Clinical Neurosciences, School of Medicine, Cardiff University, Cardiff, CF24 4HQ, UK; ^2^ Biomedicine Division, School of Biosciences, Cardiff University, Cardiff, CF10 3AX, UK; ^3^ Mary Lyon Centre, Medical Research Council, Harwell, Oxfordshire, OX11 0RD, UK; ^4^ μ-VIS X-ray Imaging Centre, Faculty of Engineering and Physical Sciences, University of Southampton, Southampton, SO17 1BJ, UK; ^5^ Biomedical Imaging Unit, Faculty of Medicine, University of Southampton, Southampton, SO16 6YD, UK

**Keywords:** Prader–Willi syndrome, Placenta, Labyrinth zone, Development, Mouse

## Abstract

The neurodevelopmental disorder Prader–Willi syndrome (PWS) is caused by paternally derived loss of gene expression from the imprinted interval on chromosome 15q11-q13. Recently, it has been suggested that the abnormal feeding-related behaviours characteristic of PWS can, in part, be developmentally programmed *in utero* via abnormal placental function. Here, we report that several PWS-associated genes were expressed in mouse placenta with three PWS-associated RNA transcripts, i.e. genes *Magel2* and *Necdin*, and the lncRNA *Sngh14*, colocalised to the *Kdr*-positive (*Kdr*+) foetal endothelial cells of the labyrinth zone central to nutrient transport. In a novel PWS mouse model (Large^+/−^) we found markedly reduced expression of PWS-associated genes in the placenta and an associated ∼25% reduction in *Kdr*+ foetal endothelial cells. Although this did not directly translate into a significant reduction in foetal growth late in gestation, these data suggest that placental function and nutrient transfer from mother to foetus could be compromised in PWS contributing to later post-natal phenotypes.

## Teaser

**Editor's choice:** Reduced expression of PWS-associated genes in the mouse placenta results in a 25% loss of the foetal endothelial cells that play a central role in nutrient and gas exchange.

## INTRODUCTION

Prader–Willi syndrome (PWS) is a neurodevelopmental disorder caused by paternally derived loss of gene expression from the imprinted cluster on chromosome 15q11-q13 (Cassidy et al., 2012). Loss of paternal gene expression primarily occurs because of large deletions spanning the imprinted interval (∼70% of cases) but can also result from maternal uniparental disomy (mUPD) or genetic variants that affect the imprinting control region (ICR) (Cassidy et al., 2012; Eggermann et al., 2023). Together, these chromosomal abnormalities and genetic variants are causal of the majority of PWS cases, and lead to complete loss of activity from all paternally expressed imprinted genes within the interval. However, there are also rare micro-deletion cases that highlight certain genes within the interval as being central to the aetiology of PWS (Fontana et al., 2017; Bieth et al., 2015; Sahoo et al., 2008).

Unsurprisingly, given the neurodevelopmental nature of PWS, all the paternally expressed genes within 15q11-q13 show high levels of expression in the brain, and much of the mechanistic research has focused on neural function in rodent and cell culture models (Gilmore et al., 2024; Zahova and Isles, 2021; Huang et al., 2021; Burnett et al., 2016). Recently, however, as well as the direct effects on brain, it has been recognised that loss of paternal gene expression may also alter other physiologies that, in turn, impact brain development and function. One hypothesis is that the metabolic and feeding problems seen in PWS may, in part, be programmed *in utero* via abnormal placental signalling (Holland et al., 2022). This fits with recent neuroimaging studies showing that structural differences in the hypothalamus of individuals with PWS are consistent across age and distinct from non-PWS obese controls (who show no structural changes), thereby suggesting a developmental origin for these brain changes (Brown et al., 2022). Interestingly, a role for PWS-associated genes and RNA transcripts (hereafter referred to as PWS genes) *in utero* has been predicted on the basis of prenatal growth restriction, with the pattern of change indicating constraint of nutrient flow during the later stages of pregnancy (Holland et al., 2022; Coan et al., 2006).

Developmental programming by maternal nutrient provision and/or placental endocrine signalling *in utero* is recognised to impact offspring brain (Isles et al., 2019; Bronson et al., 2017). Of relevance to PWS, the development of the melanocortin system – a key component of the neural control of feeding and energy homeostasis – is sensitive to changes in a multitude of endocrine and nutritional factors (Bouret, 2022). The development and connectivity of the primary constituents of the melanocortin system, pro-opiomelanocortin (POMC) and agouti-related peptide (AgRP) neurons in the hypothalamus and hindbrain, are altered in response to nutrient supply from the mother both prenatally (via the placenta) (Dearden and Ozanne, 2015) and during the early postnatal period (Bouret, 2022). For instance, maternal obesity during pregnancy causes a reduction in proliferation of neural precursor cells in the foetal hypothalamus at embryo day 13, which, in turn, leads to reduced POMC-positive neurons, and an altered hypothalamic neuropeptide profile and feeding behaviour in adult offspring (Dearden et al., 2020). The placenta directly promotes and protects foetal brain development, buffering the effects of insults, such as altered nutrient supply (Behura et al., 2019; Broad and Keverne, 2011). Consequently, it is not unreasonable to assume that loss of gene expression leading to abnormalities in PWS placental function may affect nutrient supply and/or signalling, and could be an additional factor shaping brain development and later-life feeding behaviour.

A role for PWS genes in placental function would not be unexpected as the expression of imprinted genes generally are enriched in the mouse (Higgs et al., 2023 preprint) and human placenta (Tucci et al., 2019), and genomic imprinting is thought to be intrinsically linked to the evolution of viviparity in mammals (Kaneko-Ishino and Ishino, 2019). Indeed, in early papers describing their discovery, some PWS genes have been shown to be expressed in the placenta (Boccaccio et al., 1999; Jay et al., 1997). However, there has never been a focused study of PWS gene expression in the mouse placenta. The mature mouse placenta, which develops from mid-gestation, is composed of three major layers: the maternal decidua and two foetally derived structures, i.e. the junctional zone which has a major endocrine function and the labyrinth zone which plays a major role in placental transport (John, 2024). Here, we examined PWS gene expression in the placenta across different embryonic timepoints. We showed that most PWS genes display imprinted expression in the placenta, and also examined the spatial distribution of the two PWS genes *MAGE-like protein 2* (*Magel2*), *necdin* (*Ndn*) and the long non-coding RNA (lncRNA) *Sngh14*, which includes the non-coding small nucleolar RNAs (snoRNAs) *Snord115* and *Snord116* (Plagge, 2012). All three were localised to the placental labyrinth zone and foetal endothelial cells in particular. In the recently described PWS mouse model that comprises a 3.08 Mb deletion encompassing all the paternally expressed genes in this locus (hereafter referred to as Large^+/−^) (Wolff et al., 2025 preprint), loss of expression of PWS genes impacted the cellular composition of the foetal placenta, which showed a reduction in labyrinthine endothelial cells. Together, these data suggest that loss of gene expression in the placenta of individuals with PWS have consequences for nutrient transfer across the placenta and raises the possibility of developmental programming of the developing PWS brain.

## RESULTS

### Localisation of *Magel2* to foetal endothelium of the labyrinth

**Fig. 1.**
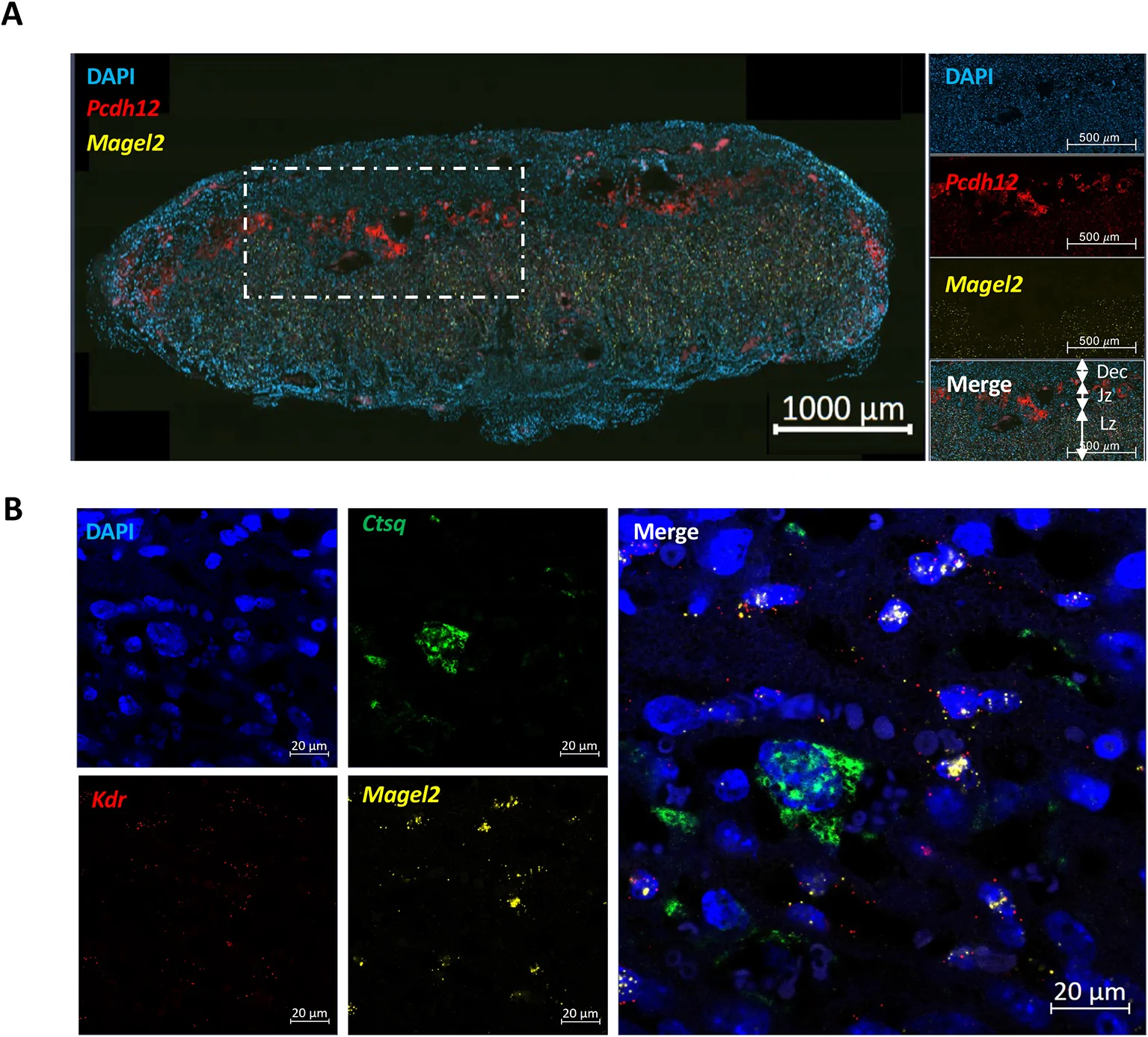
***Magel2* localisation to the foetal endothelium of the mouse placenta.** (A) Midline section of the placenta obtained from a wild-type mouse at e14.5 hybridised with RNAscope probes against *Pcdh12* (glycogen cells located in the junctional zone; red) and *Magel2* (yellow). Details of the boxed area in the main image are on the right showing individual staining as indicated. Nuclei were stained with DAPI (blue). Dec, maternal decidua; Jz, junctional zone; Lz, labyrinth zone. (B) Images show details of wild-type placenta hybridised with RNAscope probes against *Ctsq* (sinusoidal cells of the labyrinth; green), *Kdr* (foetal endothelial cells of the labyrinth; red) and *Magel2* (yellow) (B) shows that expression of *Magel2* is localised at the foetal endothelium. Nuclei were stained with DAPI (blue).

Our preliminary study focused on the PWS and Schaaf−Yang syndrome (Schaaf et al., 2013) gene *Magel2*, which has been reported previously to be expressed in the junctional zone of the placenta (Kozlov et al., 2007). We initially performed RNAscope *in situ* hybridisation assays to evaluate RNA levels of *Magel2* and *protocadherin 12* (*Pcdh12*) on midline sections obtained from placenta of wild-type mice at embryo day 14.5 (e14.5), which identifies glycogen cells located within the junctional zone (Fig. 1A) (Coan et al., 2006). Unexpectedly, the *Magel2* signal did not localise to the junctional zone but to the placental labyrinth specifically. To further define the localisation of *Magel2*, we hybridised *Magel2* together with marker genes that distinguish cell types in the labyrinth, i.e. with *cathepsin Q* (*Ctsq*) expressed exclusively by sinusoidal trophoblast giant cells and with *kinase insert domain receptor* (*Kdr*; also known as *Flk1*) specifically expressed in the foetal vascular endothelium. The *Magel2* expression signal colocalised exclusively with *Kdr*-positive (*Kdr*+) cells, indicating expression in the foetal endothelium of the placental labyrinth (Fig. 1B).

### Multiple PWS genes are expressed in mouse placenta

We then expanded our assessment of PWS genes in the placenta. By using RT-qPCR, expression levels of six genes were examined in whole placenta of wild-type mice at e10.5 and e12.5, when the mature placenta is rapidly expanding, and at e16.5, the time of maximum placental growth (Coan et al., 2006). We chose to analyse the expression of the three protein-encoding genes *necdin* (*Ndn*), *Mkrn3* and *Magel2*, and of three representative individual transcripts from the lncRNA *Snhg14*, i.e. the snoRNAs *Snord115* and *Snord116*, as well as the non-coding (nc) RNA *Ipw.* The genes were chosen because the proteins they encode have tractable cellular functions, the *Snhg14* transcripts were chosen since they are critical to the incidence of the core PWS phenotype (Sahoo et al., 2008; Skryabin et al., 2007), and because *Ipw* is a regulator of the imprinted Dlk1-DIO3 locus (Stelzer et al., 2014), which has a known role in placental function (Cleaton et al., 2016).

**Fig. 2.**
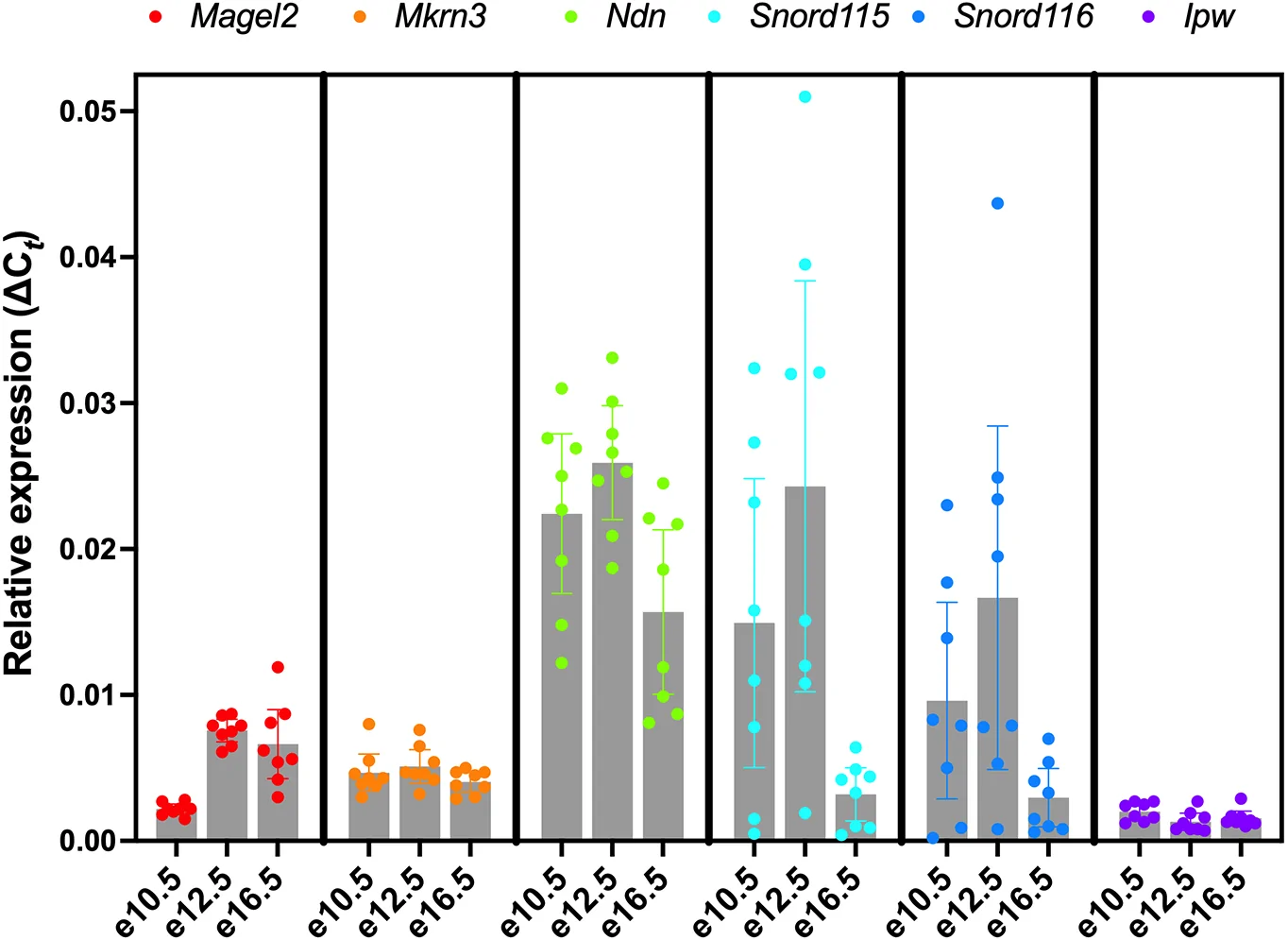
**Expression of PWS-associated genes and RNA transcripts in homogenates of placenta from wild-type at three different embryonic timepoints (e10.5, e12.5 and e16.5).** Gene expression at was normalised to the geometric mean of three housekeeping genes (see ‘Quantitative PCR’ in Materials and Methods) by using the ΔCt method. *n*=8 (4M,4F) for each timepoint. Bar graphs indicate the mean with 95% confidence intervals.

Levels of expression varied between the different PWS genes, with the highest being those of *Ndn*, *Snord115* and *Snord116*, the lowest that of *Ipw*, and those of *Mkrn3* and *Magel2* being intermediate (Fig. 2). The Angelman syndrome-associated gene ubiquitin protein ligase E3A (*Ube3a*), also showed robust expression in placenta of wild-type mice (Fig. S1A). Whereas *Mkrn3* showed relatively stable expression across all three embryonic timepoints (no main effect of embryonic timepoint; χ^2^ statistic=1.51, *P*=0.25), both *Ndn* (main effect of embryonic timepoint; χ^2^ statistic=8.66, *P*=0.013) and *Ipw* (main effect of embryonic timepoint; χ^2^ statistic=3.93, *P=*0.04) decreased in placental expression with increasing embryonic age. *Post hoc* testing (Dunn's test of multiple comparisons) revealed that for *Ndn* this change was driven by a significant decrease in expression between e12.5 and e16.5 (Z statistic=2.86, Adj. *P=*0.004), whereas *Ipw* decreased between e10.5 and e12.5, although this change was not statistically significant following Bonferroni correction for multiple testing (Z statistic=2.19, Adj. *P=*0.09). In contrast, *Magel2* placental expression increased across embryonic timepoints (main effect of embryonic timepoint; χ^2^ statistic=15.86, *P=*0.0004), with a significant increase between e10.5 and e12.5 (Z statistic=−3.75, Adj. *P=*0.0005). The snoRNA transcript levels demonstrated a pattern of expression similar to that of *Ndn*, with both *Snord115* (Z statistic=3.01, Adj. *P=*0.008) and *Snord116* (Z statistic=2.58, Adj. *P=*0.03) decreasing between e12.5 and e16.5. However, both had highly variable levels of expression, with *Snord115* (χ^2^ statistic=5.83, *P=*0.011) − but not *Snord116* (χ^2^ statistic=3.07, *P=*0.06) − demonstrating an overall main effect of the embryonic timepoint.

None of the above gene expression data demonstrated a main effect due to the sex of the animals or the interaction between embryonic timepoint and sex (timepoint−sex interaction). Full statistical details can be found in Tables S1-S3.

### Reduction in placental PWS gene expression in Large^+/−^ mice

**Fig. 3.**
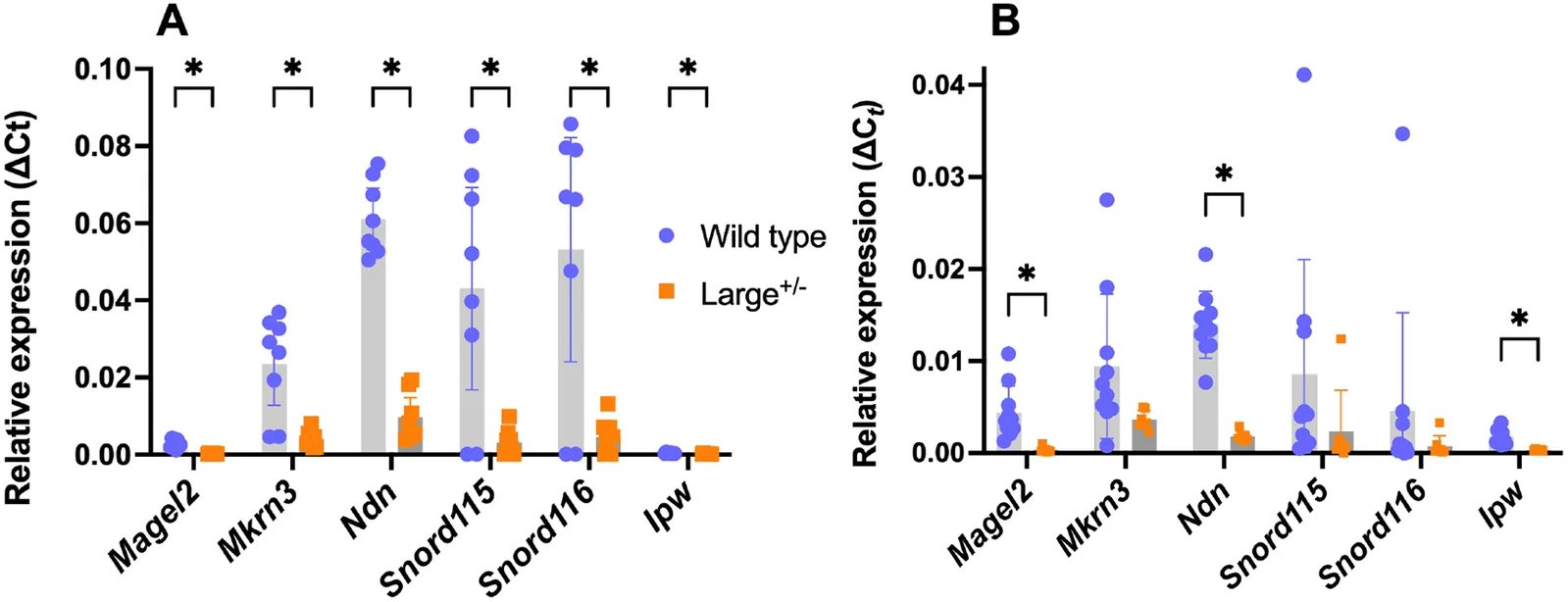
**Expression of PWS-associated genes and RNA transcripts in homogenates of placenta from wild-type and Large^+/−^ mice at two different embryonic timepoints.** (A,B) At embryonic timepoint 12.5 (e12.5) (A), expression of all PWS genes/transcripts in the placenta was significantly reduced in Large^+/−^ mice relative to wild-type controls. At e16.5 (B), although all average expression was reduced in Large^+/−^ mice, data were quite variable, and this difference only reached statistical significance for three PWS genes/transcripts (*Magel2*, *Ndn*, *Ipw*). Gene expression normalised to the geometric mean of three house-keeping genes using the ΔCt method. *n*=8 (4M,4F) for each genotype at both timepoints. Based on the Shapiro−Wilk test, normally distributed data were analysed by ANOVA factoring in genotype and sex; non-normally distributed data as determined by the Shapiro–Wilk test were analysed using Kruskal–Wallis test by factoring in genotype and sex. *P*-values indicated on the graphs are adjusted to account for multiple testing using false discovery rate (FDR) correction. Bar graphs indicate the mean with 95% confidence intervals. *P<0.05, following correction for multiple testing.

Some imprinted genes demonstrate tissue-specific imprinting (Tucci et al., 2019). We took advantage of the PWS mouse model Large^+/−^ that carries a 3.08 Mb paternal deletion encompassing all the genes in the locus (Wolff et al., 2025 preprint) to determine how placental expression of these genes is altered in response to a common PWS-associated mutation. Expression of the same six PWS genes in placenta from Large^+/−^ and wild-type littermates was examined using RT-qPCR at the two embryonic timepoints e12.5 and e16.5. At e12.5 (Fig. 3A), expression of all PWS genes was significantly reduced in Large^+/−^ mice following correction for multiple testing (see Tables S4 and S5 for full statistics). At e16.5, the picture was broadly the same, with all PWS genes showing a decrease in expression in Large^+/−^ placenta compared to wild type (Fig. 3B). However, only three of these reached statistical significance following correction for multiple testing (see Table S6 for full statistics). There was no difference in expression of the maternally expressed Angelman syndrome *Ube3a* at e12.5 and or e16.5 (Fig. S1A). There was no difference in *Ube3a* expression between males and females, and no significant interactions between genotype and sex (genotype−sex interactions) at either embryonic timepoint.

This pattern of reduced PWS gene expression matches gene expression in foetal brain of Large^+/−^ mice relative to that of wild type at the two timepoints (*n*=4 per genotype, two males and two females; Fig. S2). The same is true for expression of *Ube3a*, showing robust expression levels in foetal brain of Large^+/−^ mice, equivalent to those in foetal brain of wild-type mice (Fig. S1B).

### Localisation of *Ndn* and *Snhg14* to the foetal endothelium of the placental labyrinth

**Fig. 4.**
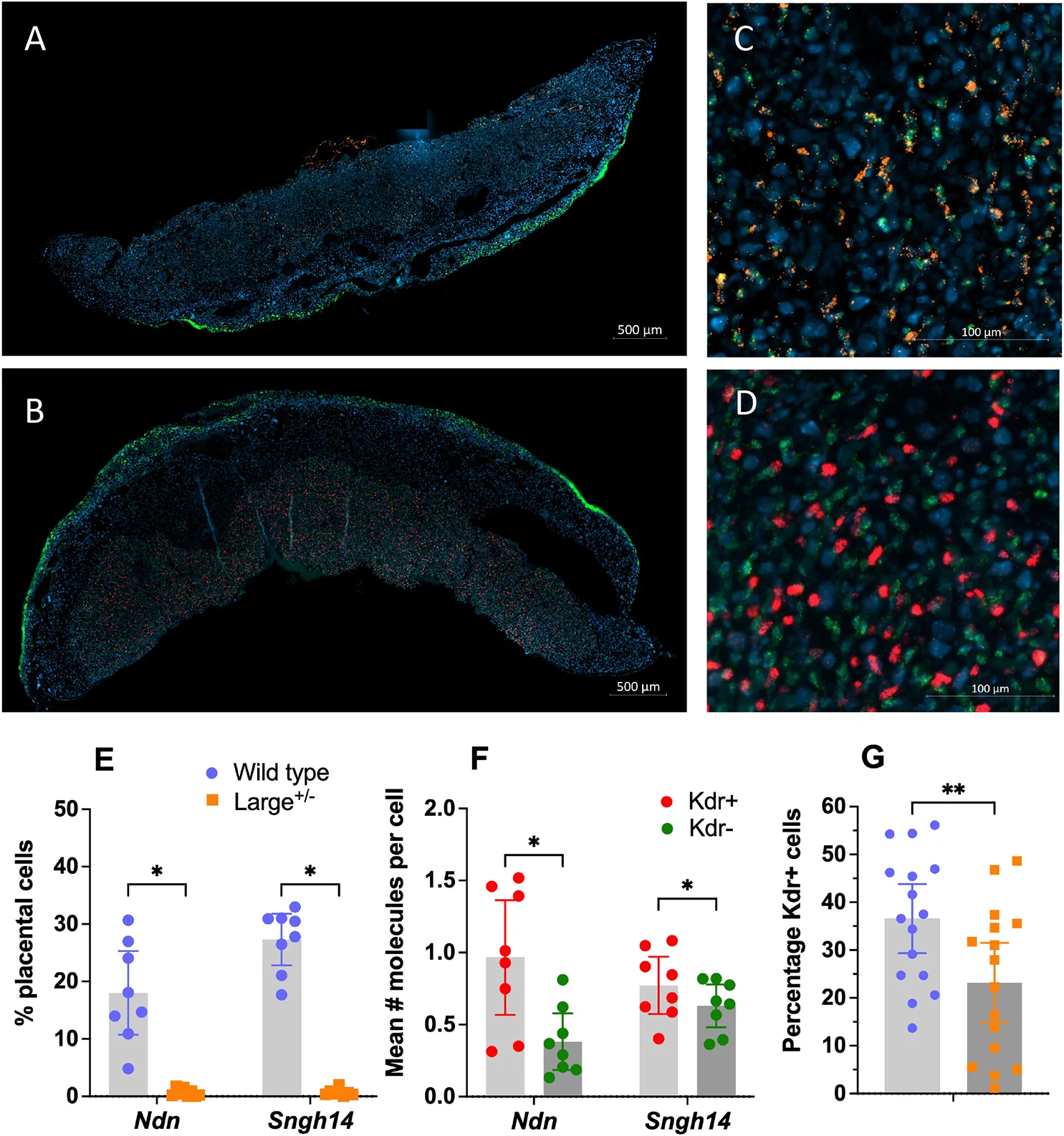
**RNAscope analysis of *Ndn* and *Snhg14* expression in endothelial cells of the placental labyrinth zone in wild-type placenta.** (A-D) Representative images of RNAscope analysis of *Ndn* (orange) *(*A) and *Snhg14* (red) (B) indicated strong expression of both genes in the labyrinth zone of the wild-type placenta. Magnified views of these RNAscope images show a high degree of co-localisation with the endothelial cell marker *Kdr* (green) (C – *Ndn*; D – *Snhg14*). Nuclei were stained with DAPI (blue). (E) Proportion of all placental cells expressing *Ndn* and *Snhg14* in placenta of wild-type and Large^+/−^ mice (Mann–Whitney test). (F) Quantification of mean number (#) of molecules per cell indicates that both genes are statistically (Wilcoxon matched-pairs signed rank test) more likely to be expressed in a *Kdr*+ than a *Kdr*− cell. (G) Quantification of total cell number and *Kdr*+ cells in demonstrates a reduction (Wilcoxon matched-pairs signed rank test) in the proportion of *Kdr*+ cells in Large^+/−^ mice (orange) relative to wild-type (blue) littermates. All bar graphs indicate the mean with 95% confidence intervals. *P<0.05; **P<0.01.

We next asked whether other PWS genes were expressed in the foetal endothelium, focusing on the most highly expressed PWS genes notably altered in the PWS model, i.e. *Ndn, Snord115* and *Snord116*. Due to technical difficulties designing probes specifically against *Snord115* and *Snord116*, we used an off-the-shelf probe against *Snhg14*, which is the lncRNA encompassing *Snord115*, *Snord116* and *Ipw* (Plagge, 2012). In wild-type placenta, expression of both *Ndn* and *Snhg14* was localised to the labyrinth zone of the placenta (Fig. 4A-D) with ∼20% of wild-type placental cells expressing *Ndn* and ∼30% expressing *Snhg14* (Fig. 4A-D). Less than 1% of placental cells in the Large^+/−^ mouse expressed these same genes (Fig. 4E) (Mann–Whitney, *Ndn P*=0.0002; *Sngh14 P*=0.0002). More detailed analysis of expression in the labyrinth demonstrated that 51% of *Ndn*-expressing, and 47% *Snhg14*-expressing cells also expressed the foetal endothelium marker *Kdr* (Fig. 4C,D). We further tested this by counting the coincidence of *Ndn* and *Snhg14* molecules with *Kdr*+ and *Kdr*− cells. The average number of molecules of *Ndn* and *Sngh14* was significantly higher in *Kdr*+ cells than that of *Kdr*− cells (Fig. 4F, Wilcoxon matched-pairs signed rank test, *P*=0.7891 for both *Ndn* and *Sngh14*).

### Loss of endothelial cells in the labyrinth in the placenta of Large^+/−^ mice

We then went on to examine if loss of expression of these and other PWS genes in Large^+/−^ placenta was associated with changes in the number of foetal endothelial cells. There was a significant reduction in the proportion of *Kdr*+ cells in the labyrinth of Large^+/−^ placenta compared to that in wild type (Fig. 4G, ANOVA F=6.878, *P*=0.014; Table S7).

### No gross difference in placenta size between Large^+/−^ and wild-type mice

Placental midline sections from Large^+/−^ (*n*=10; 6M, 4F) and wild type (*n*=13; 7 M, 6F) mice were H&E stained (*n*=4 per sample) and used to determine the midline area. There was no difference in area between wild-type and Large^+/−^ mice (Table 1).

**Table 1.** Area measurements for placental sections

Section	Mean area of placenta (μm^2^)		*P*-value		
	Wild type	Large^+/−^	Genotype	Sex	Genotype−sex interaction
1	7452843	6899598	0.356	0.368	0.769
2	7699231	7122709	0.338	0.562	0.708
19	7902259	8163069	0.325	0.191	0.976
20	8253025	7633999	0.299	0.285	0.606

The area of equivalent placenta sections was measured. Levene's test was used to determine normality of distribution (significance threshold *P*=0.05). As all data showed normally distribution; two-way ANOVA was used to determine significant differences in the area between genotype and sex or interaction between genotype and sex (genotype−sex interaction). The initial significance threshold of *P*=0.05 was reduced to *P*=0.0125 after correction for multiple testing by using the Bonferroni method.

### Foetal development in Large^+/−^ mice

As Large^+/−^ mice show almost complete lethality at birth (Wolff et al., 2025 preprint), the potential impact of abnormal placenta signalling on Large^+/−^ foetal growth was assessed at e18.5 using MicroCT. Although the volumes of whole embryos, kidney and brain were generally lower for Large^+/−^ compared to wild-type mice, none of these differences were statistically significant (Table 2). The average signal intensity [mean brightness (lower, upper 95% confidence interval)] of kidney [35,132 (32,348, 37,917)] and brain [26,095 (23,189, 29,001)] from Large^+/−^ mice was not significantly different from wild-type kidney [33,714 (30,929, 36,498), F_1,9_=0.66, *P*=0.24] or brain [26,089 (23,182, 28,995), F_1,9_=0.001, *P*=0.997] (Table 2).

**Table 2.** Whole-embryo size and selected tissue volumes in Large^+/−^ mice at e18.5

	Mean (95% CI)		F_1,9_	*P*-value	Partial *η*^2^
	Wild type (*n*=6; three females, three males)	Large^+/−^ (*n*=6; three females, three males)			
Whole embryo	1.11 cm^3^ (0.94 cm^3^, 1.28 cm^3^)	0.91 cm^3^ (0.74 cm^3^, 1.08 cm^3^)	3.36	0.100	0.272
Kidney	7.83×10^9^ μm^3^ (6.44×10^9^ μm^3^, 9.21×10^9^ μm^3^)	6.80×10^9^ μm^3^ (5.42×10^9^ μm^3^, 8.19×10^9^ μm^3^)	1.40	0.266	0.135
Brain	7.63×10^10^ μm^3^ (6.92×10^10^ μm^3^, 8.34×10^10^ μm^3^)	6.99×10^10^ μm^3^ (6.28×10^10^ μm^3^, 7.70×10^10^ μm^3^)	2.12	0.179	0.191

Reductions in whole-embryo size, and kidney and brain volumes were seen in foetuses of Large^+/−^ mice but none of these were statistically significant when compared to wild type. CI*,* confidence interval; effects size is given as partial *η*^2^.

## DISCUSSION

The idea that changes in nutrient supply and/or signalling from the placenta during development may be an additional impact on PWS brain development and function is gaining traction (Holland et al., 2022; Brown et al., 2022). Here, we explored PWS gene expression in the mouse placenta and the consequences of loss of this expression in the Large^+/−^ mouse line, which models the genetic lesion seen in ∼70% of PWS cases (Cassidy et al., 2012; Eggermann et al., 2023). We demonstrated that many PWS genes are expressed in the placenta, specifically the foetal endothelium, and that expression varies across different developmental stages. Loss of expression of PWS genes in the Large^+/−^ model (Wolff et al., 2025 preprint) impacted the cellular composition of the labyrinth zone of the placenta, which showed a reduction in the number of foetal endothelial cells. Although no detectable size difference was seen in foetuses of Large^+/−^ mice late in gestation, these data suggest that loss of gene expression in the placenta of individuals with PWS potentially have functional consequences, highlighting the need for further pre-natal studies.

The role of placenta changes across development. The endocrine lineages expand from e9.5 to e16.5, driving maternal adaptations, and the labyrinth continues to expand until term to support substantial foetal growth (Coan et al., 2006). We examined expression at key embryonic timepoints at e10.5 and e12.5 (when the mature placenta is rapidly expanding), and at e16.5 (the time of maximum placental growth), to identify whether expression of PWS genes also changed across this period. Expressions of both *Mkrn3* and the non-coding RNA *Ipw* showed little change across the timepoints examined but, in comparison, their expression was also the lowest. The highest expressed PWS transcripts, *Ndn, Snord115* and *Snord116*, all had peak expression levels at e10.5 and e12.5, decreasing significantly at e16.5. In contrast, another robustly expressed PWS gene, *Magel2*, demonstrated a significant increase in expression between e10.5 and e12.5, which was maintained at e16.5. These expression patterns across embryonic timepoints, relative levels and inferred spatial locations can also be seen when exploring a recent mouse placenta scRNA-seq database that spans mid-gestation (e7.5−e14.5) (GSE89497; Liu et al., 2018). In the placenta of the Large^+/−^ mouse model, which has a 3.08 Mb deletion encompassing all the paternally expressed genes in this locus (Wolff et al., 2025 preprint), PWS gene expression was reduced at the two timepoints assessed, indicating that these genes show a specific parent-of-origin expression − at least for the majority of cells in the placenta − with the potential to contribute to placental dysfunction in PWS.

When we explored the spatial distribution of a subset of PWS transcripts, *Magel2* – which had previously been thought to be expressed in the junctional zone (Kozlov et al., 2007) − instead colocalised with *Kdr*+ foetal endothelial cells in the labyrinth. Similarly, the most highly expressed PWS genes, *Ndn* and lncRNA *Sngh14* − which include the snoRNAs *Snord115* and *Snord116* (Plagge, 2012) − were also highly expressed in *Kdr+* foetal endothelial cells, both in terms of their coincidence and expression levels. A number of *Kdr*− cells in the labyrinth also expressed *Snhg14* but expression levels of *Ndn* and *Snhg14* were significantly higher in *Kdr*+ cells. Together, these data support a non-random distribution of PWS gene expression in the labyrinth zone.

Although we detected no gross change in overall placental morphology , counting of *Kdr*-expressing endothelial cells revealed a ∼25% reduction of the placental labyrinth zone of Large^+/−^ mice relative to wild-type controls. This is of interest because *Dlk1*, another paternally expressed imprinted gene, localises to the same cell type (Yevtodiyenko and Schmidt, 2006) further hinting at convergence of imprinted function on this cell type.

The labyrinth zone is responsible for nutrient and oxygen exchange between mother and foetus (Woods et al., 2018). In particular, the endothelial cells of the labyrinth zone play an important role in vasculogenesis, angiogenesis and maintenance of vasodilation in existing blood vessels (Sen et al., 2013; Kaufmann et al., 2004). Although placental vascularisation was not measured directly here, changes can have an impact blood flow throughout the placenta which, in turn, is vital for nutrient and oxygen transfer between mother and foetus (Sandovici et al., 2022). Small size has been reported as a feature of PWS (Yang et al., 2020; Nagai et al., 2000; Wollmann et al., 1998; Gillessen-Kaesbach et al., 1995) and the pattern of effects could reflect nutrient restriction in the later stages of pregnancy when large amounts of fat are deposited, thereby fitting with the idea of maternal and paternal imprinted genes being in conflict over resources (Haig and Wharton, 2003). When using MicroCT on a small number of foetuses, we were unable to detect a difference in foetal size between Large^+/−^ and wild-type mice at e18.5, although we recognise that studies with a higher number of foetuses are required. Interestingly, in the PWS imprinting-centre deletion mouse model, in which all PWS gene expression is also lost, surviving pups are leaner from birth (Golding et al., 2017).

A reduction in nutrient supply because of reduced endothelial cells could lead to abnormal programming of the foetus, even in the absence of clear effects on foetal growth (Sandovici et al., 2022). Certainly, developmental programming by maternal nutrient provision *in utero* is recognised to impact offspring brain (Isles et al., 2019; Bronson et al., 2017; Mikaelsson et al., 2013), including the developing melanocortin system, a key component of the neural control of feeding and energy homeostasis (Bouret, 2022). A two-hit hypothesis has been proposed, whereby loss of PWS gene expression has direct consequences for brain development but has also indirect effects via abnormal placental signalling (Holland et al., 2022). Our findings that PWS genes are expressed in the placenta and that loss of expression leads to changes in the cellular composition of the labyrinth zone suggest this is a possibility, and that nutrient transfer across the PWS placenta needs further exploration.

## MATERIALS AND METHODS

### Animals and samples

All animal studies were licensed by the Home Office under the Animals (Scientific Procedures) Act 1986 Amendment Regulations 2012 (SI 4 2012/3039), UK, and additionally approved by the Institutional Ethical Review Committees. All animal studies complied with the ARRIVE guidelines.

Wild-type placenta samples were generated from timed mating of C57BL/6J or 129SV mice (*Magel2 in situ*) within Cardiff University. The morning of plug detection was recorded as embryo day 0.5 (e0.5). Pregnant females were housed singly in cages within a vivarium maintained on a 12-h light-dark cycle (lights on from 08:00 to 20:00) at a temperature of 22±2°C and 50±10% humidity. Standard laboratory chow and water was available *ad libitum* throughout all experiments. Samples were collected between 09:00 and 11:00.

The PWS large deletion (C57BL/6J-Pcan^em4H^/H, called Large^+/−^ here) model was generated as part of the Foundation for Prader-Willi Research Pre-Clinical Animal Network (FPWR-PCAN) by the MRC Mary Lyon Centre, Harwell Oxford, UK (MRC MLC) as previously described (Wolff et al., 2025 preprint). Briefly, the large deletion (3.08 Mb) spans 2820 bp upstream of the *Mkrn3* gene and 933 bp downstream of *Ube3A*. As such, the deletion removes all the PWS-critical genes, including *Mkrn3* but excludes maternally expressed *Ube3a*. The Large^+/−^ mice were also maintained on C57BL/6J mice and were produced, genotyped, and sexed at MRC MLC.

Large^+/−^ (four males, four females) and wild-type (four males, four females) littermate samples were generated from three separate timed matings of Large^+/−^-positive male with C57BL/6J female mice. The detection of the morning a plug was recorded as e0.5. At MRC MLC, pregnant females were singly housed in IVCs (Tecniplast BlueLine 1284), on grade 4 aspen wood chips (Datesand, Stockport, UK), with shredded-paper shaving nesting material and small cardboard play tunnels for enrichment. The mice were kept under controlled light (light, 07:00–19:00; dark, 19:00–07:00), temperature (22±2°C) and humidity (55±10%) conditions. Standard laboratory chow and water was available *ad libitum* throughout all experiments. Foetal brain and placenta were collected at e12.5 and e16.5 between 09:00 and 11:00. One half of the placenta was fresh frozen on dry-ice, the other was fixed in 10% formalin for 24 h then processed and wax embedded. Brain samples from embryos were fresh frozen on dry-ice. All samples were shipped to Cardiff University for downstream processing.

Sample size was determined via Power Calculation (Lenth, 2007), and was based on difference between controls and experimental groups of 40%, with an overall standard deviation of 25%, power of 0.8 and alpha of 0.05; *n*=7 is required.

### RNA extraction and RT-PCR

Samples were transferred to lysing matrix tubes with TRI reagent (Zymo research, CA, USA) (1000 μl for brain samples, 700 μl for placenta samples). These tubes were placed in a Fast Prep® FP120 machine for 3×10 s long cycles at a speed of 5 m/s. Tubes were spun in a centrifuge at room temperature for 5 min, at a speed of 18,000 ***g***, which was used throughout. Supernatants were extracted from the lysing matrix tubes and transferred to RNase-free tubes, and an equal volume of ethanol (95−100%) was added and mixed thoroughly. RNA was then extracted using the Direct-zol™ miniprep kit as per the manufacturer's instructions (Zymo Research, CA, USA). RNA was eluted using 50 μl of DNase/RNase-free H_2_O and stored at −80°C until needed.

RNA quality and concentration was assessed using a Nanodrop™ 8000 Spectrophotometer (Thermo Fisher Scientific, MA, USA). Good quality RNA (ratio of 260/280, with a value between >1.8 and ≤2) was converted to cDNA. 2 μg of RNA was diluted in 20 µl RNase-free H_2_O and added to cDNA EcoDry™ Premix (Double Primed) (Takara Bio, CA, USA) and mixed thoroughly with a pipette. These were then subject to PCR under the following conditions: 42°C for 1 h, 70°C for 10 min. Each 20 μl sample was then diluted in 180 μl of nuclease-free H_2_O. The samples were then stored at −20°C.

### Quantitative PCR

Quantitative PCRs (qPCRs) were set up using a Corbett Robotics CAS-1200 (Qiagen, Hilden, Germany). A master mix was made up containing 12.5 μl SensiMix SYBR No-ROX Kit (Bioline, London, UK), 1.75 μl of forward and reverse primer for the chosen gene, 4 μl nuclease free H_2_O and 5 µl of sample. A no-template control (NTC) of nuclease-free H_2_O was also added for each qPCR run and all samples were run in triplicate. The PCR was run under the following conditions: 95°C for 10 min, 40 cycles of 95°C for 20 s, 60°C for 20 s, and 72°C for 20 s. Following amplification, a melt curve was generated by increasing the temperature at 1°C increments between 50°C and 99°C, and holding each change in temperature for 5 s. Sequences for primers used in this study can be seen in Table S8.

qPCR data were processed using the ΔCt method (Livak and Schmittgen, 2001) and for each tested gene the results were normalised to a geometric mean of the housekeeping genes (*Hprt1*, *Dync1li2* and *Actb*). The Levene’s test was used to test normality of the data. If normally distributed, data were analysed by two-way ANOVA of the ΔCt values. For the analysis of wild-type PWS gene expression across development, the factors ‘embryonic timepoint’ (i.e. e10.5, e12.5, e16.5) and ‘sex’ (male, female) were applied. To analyse Large^+/−^ data, the factors ‘genotype’ (wild type, Large^+/−^) and ‘sex’ (male, female) were used. For non-normally distributed data, a log transformation was first carried out to see if this would cause the data to assume normality, if the data were still not normally distributed Kruskal−Wallis test was used as a non-parametric equivalent significance test. Both tests used significance thresholds of *P*<0.05. *P*-values were then adjusted to account for multiple testing using False Discovery Rate (FDR) correction.

### Image analysis of H&E-stained placentas

Haematoxylin and eosin (H&E)-stained placenta sections from wild-type and Large^+/−^ mice were imaged, and measured using the Zen image analysis tool [Zeiss Zen (blue edition), Zeiss, Oberkochen, Germany]. These area values were then analysed by using RStudio and Levene’s test to check for normality and two-way ANOVA with the factors genotype (wild type, Large^+/−^) and sex (male, female) to determine a difference in placenta areas. Both tests used initial significance thresholds of *P*<0.05. The Bonferroni method was used to correct *P*-values for multiple testing before determining whether a result was significant.

### RNAscope assays

RNAscope® Multiplex Fluorescence Assay was performed according to the manufacturers guidelines essentially as previously described by Tunster et al. (2018), using RNAscope® Multiplex Fluorescence reagent kits (Advanced Cell Diagnostics, Newark, CA, USA). All probes listed in as follows were from Advanced Cell Diagnostics. For *Magel2* localisation, e14.5 midline sections of placenta from wild-type 129/Sv mice were hybridised with probes against *Pcdh12* (Mm-Pcdh12-489891-C1, cat. no.: 489891; 1:500) and *Magel2* (Mm-Magel2-C3, cat. no.: 502971-C3; 1:1000) or probes against *Ctsq* (sinusoidal cells of the labyrinth; Mm-Ctsq-423681-C1, cat. no.: 423681; 1:500); *Kdr* (foetal endothelial cells of the labyrinth; Mm-Kdr-C2, cat. no.: 414818-C2) and *Magel2*. For quantification of foetal endothelium and localisation of *Ndn* and *Snhg14* wild-type and Large^+/−^ e16.5 BL6 midline placental sections were hybridised with probes against *Kdr* and either *Ndn* (Mm-Ndn-C3, cat. no.: 442711-C3) or *Snhg14* (Mm-Snhg14-C3, cat. no.: 1041151-C3). *Snhg14*, the long non-coding RNA (lncRNA) comprising *Snord115*, *Snord116* and *Ipw*, was chosen as it was not possible to design probes against the individual small nucleolar RNAs (snoRNAs) and non-coding RNAs (ncRNAs); *n*=8 placenta of each genotype with two sections counted per placenta. Each slide contained two placenta sections, one was treated with probes against the aforementioned genes and RNA transcripts (termed the ‘probe section’), the other with probe dilutant as control to distinguish between true and background signal (Fig. S3).

### RNAscope image analysis

Slides were imaged using the Zeiss fluorescence microscope, with each channel for each group being set to the same light exposure times. Images were taken within 1 week of the slide being produced. Images were then processed using Zeiss Zen (blue edition) imaging software. Background subtraction and Gauss settings were used to pre-process the images, before a background fluorescence value was calculated from an adjacent no-probe control section on the slide. Only signal intensity above this no-probe threshold value was deemed ‘true signal’. The analysis gave the number of positive cells for each gene within each placenta section, and the number of true-signal molecules found within each positive cell. The proportion of cells showing true signals within each placenta section for each tested gene was then calculated, as well as the proportion of cells showing colocalization, and the data were compared for wild-type and Large^+/−^ mice. For the percentage of *Kdr*+ (endothelial) cells per placenta, an arcsine square root transformation was first used, followed by a two-way ANOVA to determine the presence of statistically significant effects of the factors genotype (wild type, Large^+/−^) and sex (male, female). For the wild-type placenta PWS gene (*Snhg14* or *Ndn*) molecule count data, the Mann–Whitney and Wilcoxon tests were used to determine if there was a significant difference between cell type − either *Kdr*+ (endothelial) or *Kdr*− (non-endothelial) − because the obtained data were non-parametric and unpaired. All statistical tests for this step were performed using RStudio (https://posit.co/products/open-source/rstudio) and *P*<0.05, before correction for multiple testing by using the Bonferroni method.

### MicroCT

Six Large^+/−^ and six wild-type embryos (three males, three females for each genotype, derived from two separate litters) were dissected into ice-cold phosphate-buffered saline (PBS) and exsanguinated by severing the umbilical vessels. After washing in PBS, embryos were fixed by immersion in 4% paraformaldehyde (PFA) in PBS for 7 days at 4°C, before storing in 1% PFA at 4°C.

Embryos were contrasted by immersion in 50% Lugol's iodine solution (1:1 Lugold:ddH_2_O) for 2 weeks, protected from light, and solution was exchanged for fresh one every 2 days. After contrasting, samples were washed in ddH_2_O for at least 1 h before embedding in an acrylic mount in 1% agarose dissolved in ddH_2_O and allowed to set for at least 2 h.

MicroCT data sets were acquired using a Skyscan 1272 scanner (Bruker, Billerica, MA, USA) with the X-ray source set to 70 kV and using 1-mm aluminium filter. Pixel resolution of 15 μm/pixel was set at 3×3 camera pixel binning. Four projections were acquired and averaged every 0.4° through a total rotation of 360° with a final step of 25 random movements. NRecon (Bruker) was used for 3D reconstruction to produce a final volume in a PNG image-stack format.

For image analysis, each MicroCT dataset was imported into the commercial software Avizo (Avizo 3D 2024.2, Thermo Fisher). The whole embryo, brain and kidney were manually segmented using Avizo's segmentation editor. The organ volume for each embryo was normalised to total embryo volume to enable a direct comparison. The average intensity (note that the tissue was iodinated) of the organs was measured by masking the organs of the 16 bit depth volume and summing all the pixel intensities in the mask divided by the volume of the mask. The segmented organ volume was used to mask each organ from the total embryo.

Although both males and females were included in sampling, we were underpowered to detect genotype−sex interactions and, therefore, chose to use ANCOVA to analyse the data, with ‘genotype’ as a fixed factor and ‘sex’ as a co-factor. All statistical tests for this step were performed in SPSS and a *P*-value of 0.05 was used, before correction for multiple testing using the Bonferroni method.

### Statistics

The statistical analysis approach for each specific experimental method is provided in the sub-sections above. Generally, if the data were normally distributed (Shapiro–Wilk test), one- or two-way ANOVA or analysis of covariance (ANCOVA) tests were used. For these analyses, the F-value (and associated degrees of freedom) and *P*-value are reported. For non-parametric data with mutiple factors, Kruskal–Wallis test was used as an equivalent. Here, the χ^2^ statistic and *P*-values were reported. For other non-parametric analyses, Mann–Whitney or Wilcoxon matched-pairs signed rank test was used.

## Supplementary Material

10.1242/dmm.052833_sup1 Supplementary information
