# Supplementary material for: Placental defects revealed by modelling Prader–Willi syndrome in mice
Source: Dis Model Mech. 2026 Sep 1;19(8):dmm052833. doi: 10.1242/dmm.052833 (PMC13580601; doi:10.1242/dmm.052833)
Supplement: Supplementary file 1 [file dmm-19-052833-s1.pdf]

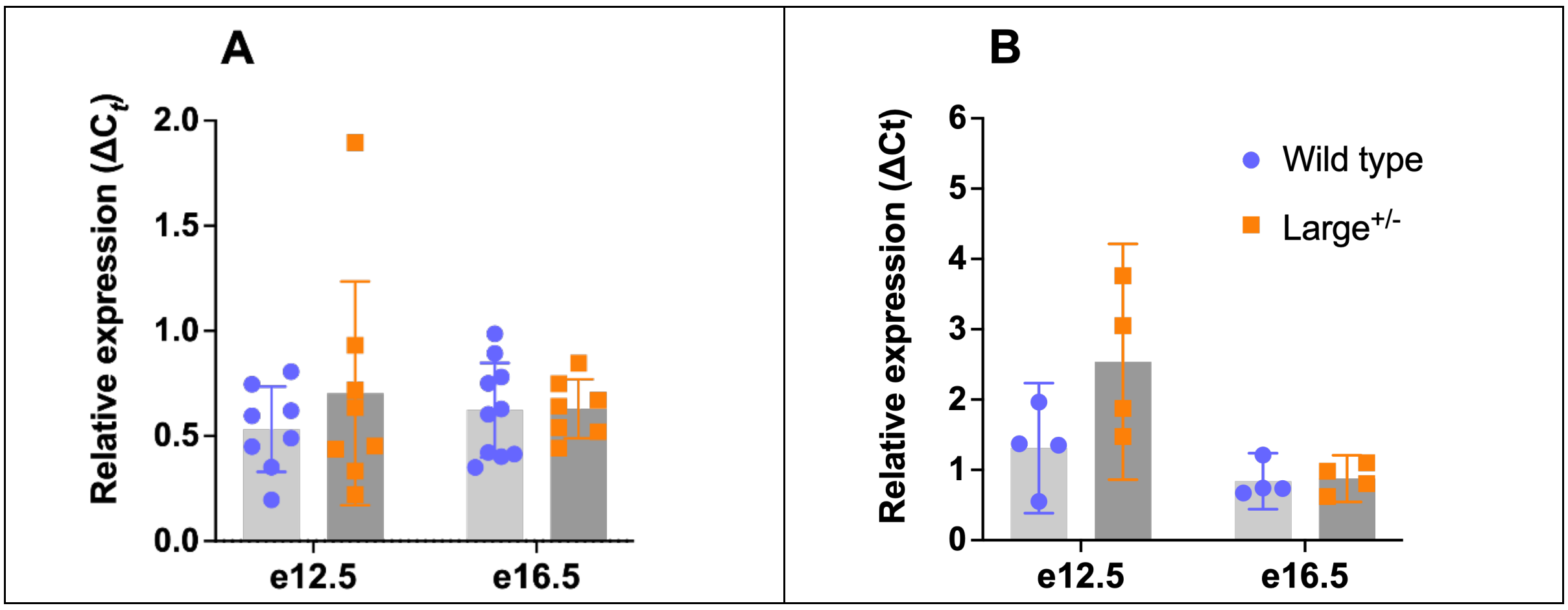

Fig. S1. Expression of *Ube3a* in placenta (A) and whole brain (B) homogenate from wild-type and Large$^{+/-}$ mice at two different embryonic timepoints. Gene expression normalised to the geometric mean of three house-keeping genes using the ΔCt method. Bars are means with 95% CI indicated.

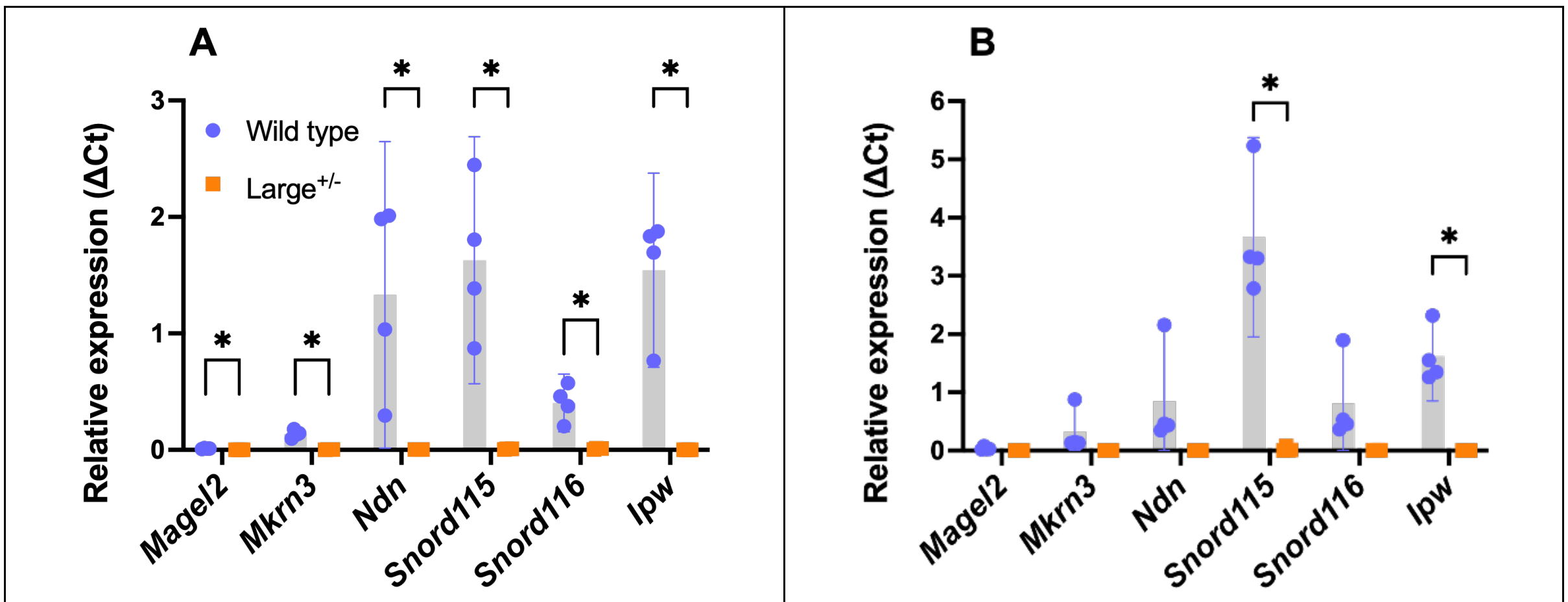

Fig. S2. Expression of PWS genes in whole brain homogenate from wild-type and Large$^{+/-}$ mice at two different embryonic timepoints. At e12.5 (**A**), expression of all PWS genes in the placenta was significantly reduced in Large$^{+/-}$ mice relative to wild-type controls. At e16.5 (**B**), although means expression was reduced in Large$^{+/-}$ mice, this only reached statistical significance for two PWS genes. Gene expression normalised to the geometric mean of three house-keeping genes using the ΔCt method. Bars are means with 95% CI indicated.

Disease Models & Mechanisms • Supplementary information

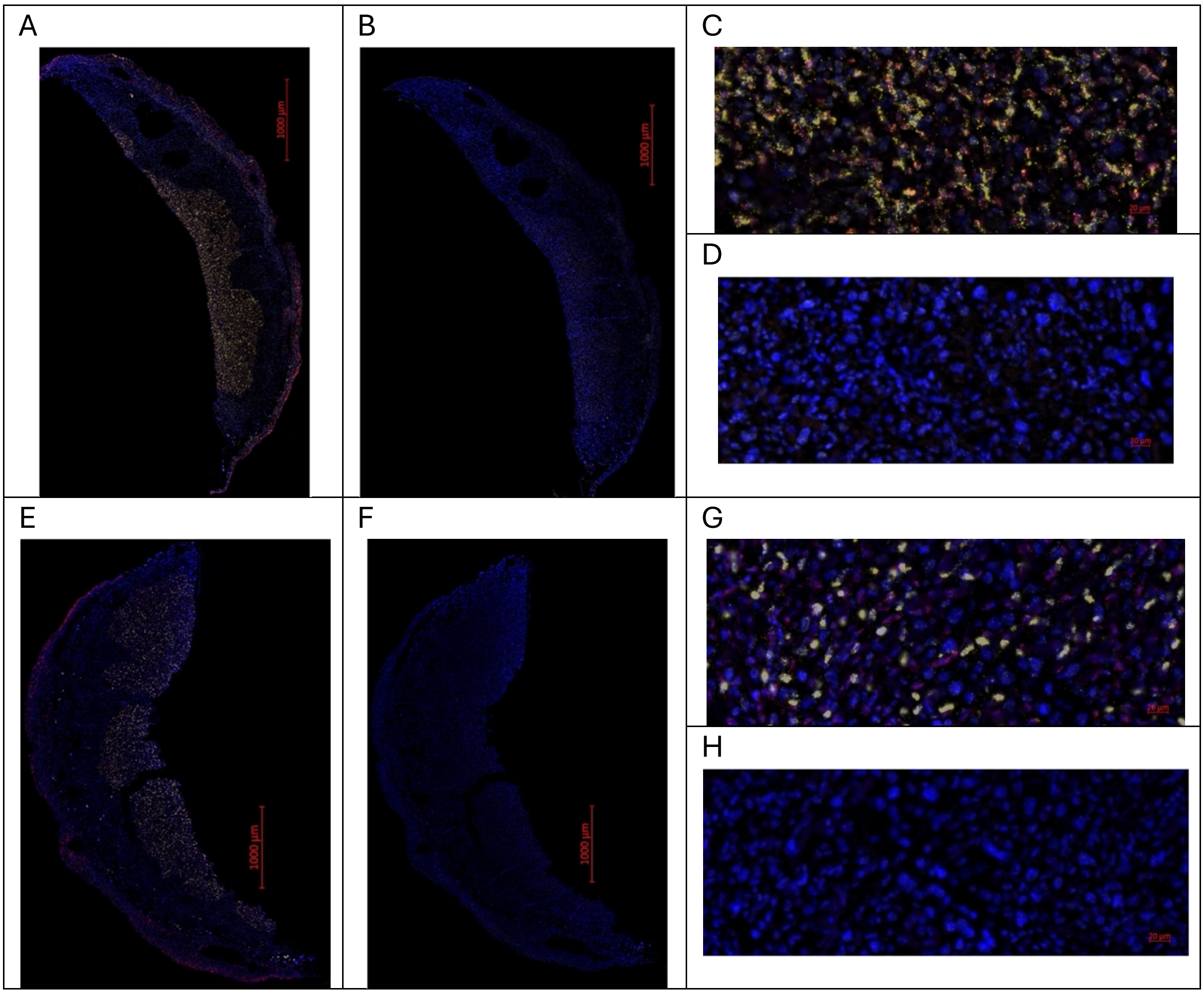

Fig. S3. Representative images of RNAscope analysis of *Ndn* (A and C) and *Snhg14* (E and G) and the endothelial cell marker *Kdr* in wild type placenta. No-probe control images for *Ndn/Kdr* (**B** and **D**) and *Snhg14/Kdr* (**F** and **H**) illustrate the level of background fluorescence for each sample that was accounted for in the image analysis.

Disease Models & Mechanisms • Supplementary information

Table S1. Statistics for two-way ANOVA analysis of PWS gene expression in wild type placenta across three embryonic timepoints (e10.5, e12.5 and e16.5). Based on the results of the Shapiro-Wilk test, normally distributed data were analysed by ANOVA using the factors TIMEPOINT and SEX. Statistically significant differences are highlighted in bold.

	Factors investigated via two-way ANOVA test					
	SEX		TIMEPOINT		SEX*TIMEPOINT	
**Gene name**	**F value**	**P-value**	**F value**	**P-value**	**F value**	**P-value**
Mkrn3	1.44	0.25	1.51	0.25	0.97	0.40
Ube3a	0.23	0.64	5.67	**0.012**	0.48	0.62
Ipw	1.96	0.18	3.93	**0.04**	2.61	0.10
Snord115	0.002	0.97	5.83	**0.011**	1.47	0.26
Snord116	0.14	0.71	3.07	**0.07**	1.84	0.19

Table S2. Statistics for Kruskal-Wallis analysis of wild-type placental PWS gene expression in wild type placenta across three embryonic timepoints (e10.5, e12.5 and e16.5). Non-normally distributed data as determined by the Shapiro-Wilk test, were analysed by Kruskal-Wallis using the factors TIMEPOINT and SEX. Statistically significant differences are highlighted in bold.

Gene name	Kruskal-Wallis test; TIMEPOINT		Kruskal effect size value	Kruskal-Wallis test; SEX		Kruskal effect size value
	χ2 statistic	P-value		χ2 statistic	P-value	
Magel2	15.86	**0.0004**	0.66	0.003	0.954	-0.045
Ndn	8.66	**0.013**	0.317	1.47	0.225	0.021

Table S3. Results of *post-hoc* Dunn pairwise tests on wild type PWS genes placental gene expression across three embryonic timepoints, including the z statistic and the Bonferroni corrected p-value. Statistically significant differences are highlighted in bold.

Gene name	E10.5 – E12.5		E10.5 – E16.5		E12.5 – E16.5	
	Z statistic	Adjusted p-value	Z statistic	Adjusted p-value	Z statistic	Adjusted p-value
Mkrn3	-0.88	1	0.57	1	1.45	0.44
Magel2	-3.75	**0.0005**	-3.04	**0.007**	0.707	1
Ndn	-0.85	1	2.02	0.1	2.86	**0.004**
Snord116	-0.92	1	1.66	0.29	2.58	**0.03**
Ipw	2.19	0.09	1.2	0.7	-0.99	1
Snord115	-1.03	0.9	1.98	0.1	3.01	**0.008**

Disease Models & Mechanisms • Supplementary information

Table S4. Statistics for two-way ANOVA analysis of PWS gene expression in wild type and *Large*$^{+/-}$ placenta at e12.5. Based on the results of the Shapiro-Wilk test, normally distributed data were analysed by ANOVA using the factors GENOTYPE and SEX. Statistically significant differences are highlighted in bold.

	Factors investigated via two-way ANOVA test					
	GENOTYPE		SEX		GENOTYPE*SEX	
Gene name	F value	P-value	F value	P-value	F value	P-value
Magel2	91.16	**5.89E-07**	0.49	0.498	3.46	0.088
Snord115	59.19	**1.17E-04**	0.70	0.695	1.17	0.314
Ipw	14.70	**0.0024**	0.29	0.287	0.03	0.858

Table S5. Statistics for Kruskal-Wallis analysis of wild-type placental PWS gene expression in wild type and *Large*$^{+/-}$ placenta at e12.5. Non-normally distributed data as determined by the Shapiro-Wilk test, were analysed by Kruskal-Wallis using the factors GENOTYPE and SEX. Statistically significant differences are highlighted in bold.

Gene name	Kruskal-Wallis test; GENOTYPE		Kruskal effect size value	Kruskal-Wallis test; SEX		Kruskal effect size value
	χ2 statistic	P-value		χ2 statistic	P-value	
Mkrn3	7.5	**0.0063**	0.461	0.10	0.753	-0.064
Ndn	10.5	**0.0012**	0.679	0.05	0.817	-0.068
Snord116	7.5	**0.0062**	0.464	2.70	0.100	0.121

Disease Models & Mechanisms • Supplementary information

Table S6. Statistics for two-way ANOVA analysis of PWS gene expression in wild type and *Large*$^{+/-}$ placenta at e16.5. Based on the results of the Shapiro-Wilk test, normally distributed data were analysed by ANOVA using the factors GENOTYPE and SEX. Statistically significant differences are highlighted in bold.

	Factors investigated via two-way ANOVA test					
	GENOTYPE		SEX		GENOTYPE*SEX	
**Gene name**	**F value**	**P-value**	**F value**	**P-value**	**F value**	**P-value**
Magel2	62.698	**2.5E-06**	0.839	0.376	0.789	0.391
Snord115	4.511	0.053	0.200	0.662	1.892	0.192
Snord116	0.936	0.351	0.263	0.167	1.520	0.239
Ndn	215.593	**1.8E-09**	0.146	0.708	0.340	0.570
Mkrn3	2.734	0.122	0.062	0.808	0.001	0.979
Ipw	57.387	**4.0E-06**	0.636	0.439	0.016	0.900

Table S7. Total cell counts for placenta (DAPI stained cells) and endothelial cell (*Kdr*-positive) broken down by genotype and sex. The individual counts were used to derive the percentage *Kdr*-positive cell values used in Figure 4G of the Main text.

		Average total cell counts (±SD)	Average *Kdr*-positive cell counts (±SD)
Wild type placenta	Male	23,259 (5,885)	10,040 (687)
	Female	23,323 (4,400)	8,448 (2807
Large$^{+/-}$ placenta	Male	22,918 (5,048)	4846 (3436)
	Female	22,207 (5,973)	4835 (754)

Disease Models & Mechanisms • Supplementary information

Table S8. Primer sequences and concentrations used in qPCR

Gene Primer		5'-3' Sequence	Optimum Conc. (nM)
Mrkn3	For	CCAATCAGTTGCTTAAGAAGTTGC	300
	Rev	AAGAGCCAACGGTCATCAGAG	300
Magel2	For	GCATAGCAAGCCAGCCTCAG	300
	Rev	GTAGACGAGCCTGTGGAGCCT	50
Necdin (*Ndn*)	For	ATGGTGCAGAAGCATCCTCAG	300
	Rev	ATGGTGTGGAGATTGGTCAGC	300
Snord116	For	TGGATCTATGATGATTCCCAG	300
	Rev	TGGACCTCAGTTCCGATGAG	50
Ipw	For	TGCTGTTATTGCCTTGCCTG	300
	Rev	TGGTGAAGCTGCTGGTAGAA	300
Snord115	For	ACAACCCACTGTCATGAAGAAAGG	50
	Rev	CCTCAGCGTAATCCTATTGAGCAT	700
Ube3a	For	CAGACGTGACCATATTATAGATGATGC	300
	Rev	CCACATACAACTGCTTCTTCAAGTCT	300
Hprt	For	GCGATGATGAACCAGGTTATGA	300
	Rev	GCCTCCCATCTCCTTCATGA	300
Dynein	For	GACCTCAGGCTCAGACGAAGAC	300
	Rev	AAGACGCTCATGGCATCACA	300
Beta-actin	For	TTCTGGTGCTTGTCTCACTGA	300
	Rev	CAGTATGTTCGGCTTCCCATTC	300
